# Joint Degradation in a Monkey Model of Collagen-Induced Arthritis: Role of Cathepsin K Based on Biochemical Markers and Histological Evaluation

**DOI:** 10.1155/2016/8938916

**Published:** 2016-02-02

**Authors:** Makoto Tanaka, Hiroyuki Yamada, Satoshi Nishikawa, Hiroshi Mori, Yasuo Ochi, Naoto Horai, Minqi Li, Norio Amizuka

**Affiliations:** ^1^Research Promotion, Ono Pharmaceutical Co., Ltd., Shimamoto, Osaka 618-8585, Japan; ^2^Discovery Research Laboratories, Ono Pharmaceutical Co., Ltd., Shimamoto, Osaka 618-8585, Japan; ^3^Shin Nippon Biomedical Laboratories, Ltd., Drug Safety Research Laboratories, Miyanoura, Kagoshima 891-1394, Japan; ^4^Department of Developmental Biology of Hard Tissue, Graduate School of Dental Medicine, Hokkaido University, Sapporo 060-8586, Japan; ^5^Shandong Provincial Key Laboratory of Oral Biomedicine, The School of Stomatology, Shandong University, Jinan 250012, China

## Abstract

The role of cathepsin K in joint degradation in a model of collagen-induced arthritis (CIA) in cynomolgus monkey was examined using biochemical markers and histology. Joint swelling, urinary C-telopeptide of type II collagen (CTX-II), deoxypyridinoline (DPD), and N- and C-telopeptides of type I collagen (NTX and CTX-I, resp.) were analyzed. Immunohistochemistry of type II collagen, cathepsin K, and CTX-II were performed using joints. Joint swelling reached peak on day 42 and continued at this level. The CTX-II level peaked on day 28 and declined thereafter, while CTX-I, NTX, and DPD reached plateau on day 43. Joint swelling was positively correlated with CTX-II increases on days 20 and 42/43, with increases in CTX-I and NTX/Cr on days 42/43 and 84, and with DPD increases throughout the study period. Intense cathepsin K staining was observed in osteoclasts and in articular cartilage and synovial tissue in arthritic joints. CTX-II was present in the superficial layer of articular cartilage in CIA monkeys. Evidence from biochemical markers suggests that matrix degradation in the CIA model starts with degradation of cartilage, rather than bone resorption. Cathepsin K expressed in osteoclasts, articular cartilage, and synovial tissue may contribute to degradation of cartilage.

## 1. Introduction

Rheumatoid arthritis (RA) is characterized by chronic inflammation of synovial joints leading to periarticular bone loss/erosion and cartilage destruction, which then cause reduced function and poorer quality of life [1, 2]. Bone loss is caused by a relative increase in bone resorption mediated by osteoclasts over bone formation mediated by osteoblasts [3]. Elevation of inflammatory cytokines such as tumor necrosis factor alpha and interleukin-1 promotes cell differentiation of the Th17 cell subset and induces osteoclastogenesis in RA [4]. Antiresorption agents are effective in preventing bone loss, but not disease suppression, in clinical studies of RA [5, 6]. Cartilage degradation appears to be a result of proteolysis of extracellular matrix. Matrix metalloproteinases have been considered as a potent target for the treatment of RA, but the therapeutic efficacy of matrix metalloproteinase inhibitors is not verified.

Cathepsin K, a member of the papain cysteine protease superfamily, is released by osteoclasts and degrades type I collagen of bone [7]. N-terminal and C-terminal telopeptide of type I collagen (NTX and CTX, resp.) are generated by cathepsin K during the bone resorption process and are used as biochemical markers of osteoporosis [8, 9]. Genetic evidence also suggests a critical role of cathepsin K in bone resorption in humans and mice [10, 11]. Therefore, many cathepsin K inhibitors have been developed and are likely to be the next generation of therapy for bone resorption diseases such as osteoporosis [12–14].

Cathepsin K is also expressed in synoviocytes and chondroclasts [15–17], and spontaneous synovitis and cartilage degradation occur in cathepsin K-overexpressing mice, in addition to histological changes in joints [18]. A cartilage marker, CTX-II, is a predictor of an increased risk of radiological progression in early RA [19] and is generated by cleavage of type II collagen by proteolytic enzymes, including cathepsin K [20]. Furthermore, a cathepsin K inhibitors reduced the urinary CTX-II level in patients with osteoporosis [21] and showed a cartilage protective effect in several models of RA and osteoarthritis [22–25].

Rat models induced by immunization with type II collagen (CIA) or adjuvant are well known as animal models for RA [26]. CIA develops through an autoimmune response to a connective tissue component and has advantages over bacterial arthritis models [27]. However, the CIA model in rats is difficult to extrapolate to humans. First, there are no changes in axial joints in the rat CIA model, but a periosteal reaction is observed. Second, joint swelling in the model is transient and spontaneously recovered. Third, bones of rats continuously grow over the lifespan, unlike other animals [28]. Nonhuman primates have the closest skeletal similarity to humans, and monkey CIA model has come to be used as a RA model to evaluate cross-reactivity in humans for development of drugs such as antibodies [29, 30]. Skeletal maturation and bone turnover in monkey are generally considered to most closely resemble human, and therefore monkeys are often used as a model for osteoporosis [31, 32]. In addition, symptoms of monkey CIA model are irreversible and persistent for a long time like RA. On the other hand, analyses of cartilage and bone turnover markers and histological evaluation in the monkey CIA model have not been performed in detail.

In this study, we evaluated bone and cartilage degradation based on biochemical markers and histological evaluation in the monkey CIA model. X-ray is commonly used for diagnosis and follow-up in RA patients, but there are not enough researches to assess human cartilage in clinical studies since it is difficult to observe cartilage by X-ray. Longitudinal changes in bone resorption markers (urinary CTX-I, NTX, and deoxypyridinoline (DPD)) and a cartilage marker (urinary CTX-II) and correlations of the levels of these markers with joint swelling were analyzed. In the histological evaluation, proximal interphalangeal joints from 6 CIA monkeys with joint destruction and 3 normal monkeys were analyzed using immunohistochemistry for type II collagen, cathepsin K and CTX-II, and electron microscopy.

## 2. Methods

### 2.1. Animals

Female cynomolgus monkeys (*Macaca fascicularis*) aged 3 to 5 years were obtained from Gaoyao Kangda Laboratory Animals Science & Technology Co., Ltd. (Gaoyao, China), Guangzhou Kesen Imports & Exports Co., Ltd. (Guangzhou, China), Guangxi Grandforest Scientific Primate Company, Ltd. (Guangxi, China), and Guangdong Scientific Instruments & Materials Import/Export Corporation (Guangzhou, China). All procedures for animal care and experimentation were approved by the Institutional Animal Care and Use Committee of SNBL and were performed in accordance with standards published by the National Research Council (Guide for the Care and Use of Laboratory Animals, NIH OACU) of the National Institutes of Health Policy on Human Care and Use of Laboratory Animals. Additionally, the animals used in this model received special treatment to moderate emaciation. In accordance with these standards, every effort was made to ensure that the animals were free of pain and discomfort.

### 2.2. Induction of Arthritis

Bovine type II collagen solution (4 mg/mL, Collagen Research Center, Tokyo, Japan) and Freund's complete adjuvant (Becton Dickinson, Grayson, GA, USA) were mixed in equal proportions using a syringe. Each monkey was intracutaneously immunized with 2 mL of the emulsion on the back under anesthesia by intramuscular injection of 10 mg/kg ketamine. A second immunization with type II collagen was conducted 3 weeks later in the same manner [33, 34].

### 2.3. Joint Swelling Score

Arthritis level was evaluated by monitoring the degree of swelling in 64 joints: the metacarpophalangeal, proximal interphalangeal, and distal interphalangeal joints, and the wrist, ankle, elbow, and knee [34]. Each joint was assessed using the following evaluation criteria: 0, no abnormality; 1, swelling not visible but can be determined by touch; 2, swelling slightly visible and can be confirmed by touch; 3, swelling clearly visible; and 4, rigidity of the joints. The joint swelling score for each animal was designated as the total score over all joints.

### 2.4. Measurement of Urinary Cartilage and Bone Turnover Markers

Urine trays were attached to cages and approximately 24-hour cumulative urine was collected. The collected urine was centrifuged and the supernatant was stored at −80°C. Urinary CTX-I, CTX-II, DPD, and NTX were measured by ELISA using Urine BETA CrossLaps (Immunodiagnostic Systems, Boldon, UK), urine Pre-CartiLaps (Nordic Bioscience A/S), Pyrilinks-D (Metra Biosystems, Mountain View, CA), and Osteomark (Ostex International, Seattle, WA) kits, respectively. Urinary CTX-I, CTX-II, NTX, and DPD values were corrected for the urinary creatinine (Cr) concentration measured with an autoanalyzer (Hitachi 7080, Hitachi, Tokyo, Japan) [35].

### 2.5. Protocol for Study of Joint Swelling and Urinary Cartilage and Bone Turnover Markers

Urinary biomarkers were measured in 12 CIA monkeys. Swelling was monitored before immunization and on days 20, 30, 35, 42, 56, 70, and 84. Urine samples were collected before immunization and on days 20, 30, 43, and 84.

### 2.6. Protocol for Study of Histological Evaluation

Histological examination was conducted in 6 CIA monkeys and 3 normal monkeys. Swelling was monitored before immunization and on days 21, 35, 49, and 63. Urinary CTX-I/Cr, DPD/Cr, and CTX-II/Cr were measured before immunization and on days 20, 34, 48, and 62. Monkeys were sacrificed by intravenous injection of a solution of sodium pentobarbital (Tokyo Chemical Industry Co., Ltd., 64.8 mg/mL, 0.4 mL/kg) and necropsied. The skin around the carpal region and the proximal interphalangeal (PIP) joints were excised from the left forelimb. Through opened minute holes in the articular capsule, the PIP joint of the second finger was fixed with 4% paraformaldehyde in 0.1 M phosphate buffer (pH 7.4) for approximately 24 h at 4°C. Specimens were then demineralized with 10% ethylenediaminetetraacetic acid disodium (EDTA) solution and dehydrated with increasing concentrations of ethanol before being embedded in paraffin. The decalcified joints were cleaved sagittally and embedded in paraffin. Sagittal sections were then cut and stained with hematoxylin and eosin (H&E) and immunohistochemically for detection of type II collagen, cathepsin K, CTX-II, and tissue nonspecific alkaline phosphatase (ALP) [36–38]. The sections were also used for tartrate-resistant acid phosphatase (TRAP) enzyme histochemistry.

### 2.7. Transmission Electron Microscopy (TEM)

For electron microscopy, fixed specimens were decalcified, postfixed with OsO_4_, dehydrated, and embedded in epoxy resin (Epon 812, Taab, Reading, UK). Ultrathin sections from nondecalcified samples were obtained using a Sorvall MT-5000 microtome (Ivan Sorvall Inc., Norwalk, CT, USA) with a diamond knife's boat filled with ethylene glycol. TEM observation was conducted at 80 kV using a Hitachi H-7100 electron microscope (Hitachi Co. Ltd, Tokyo, Japan) [39].

### 2.8. Statistical Analysis

Correlations between joint swelling and increases in urinary CTX-II/Cr, CTX-I/Cr, NTX/Cr, and DPD/Cr were analyzed by Spearman rank correlation test using GraphPad Prism ver. 5.0 (San Diego, CA, USA). The correlation analyses were conducted using overall data during study period based on the area under the concentration/score curve (AUC). Correlations of biomarker levels and joint swelling were analyzed at all time points.

## 3. Results

### 3.1. Biochemical Markers

**Figure 1:**
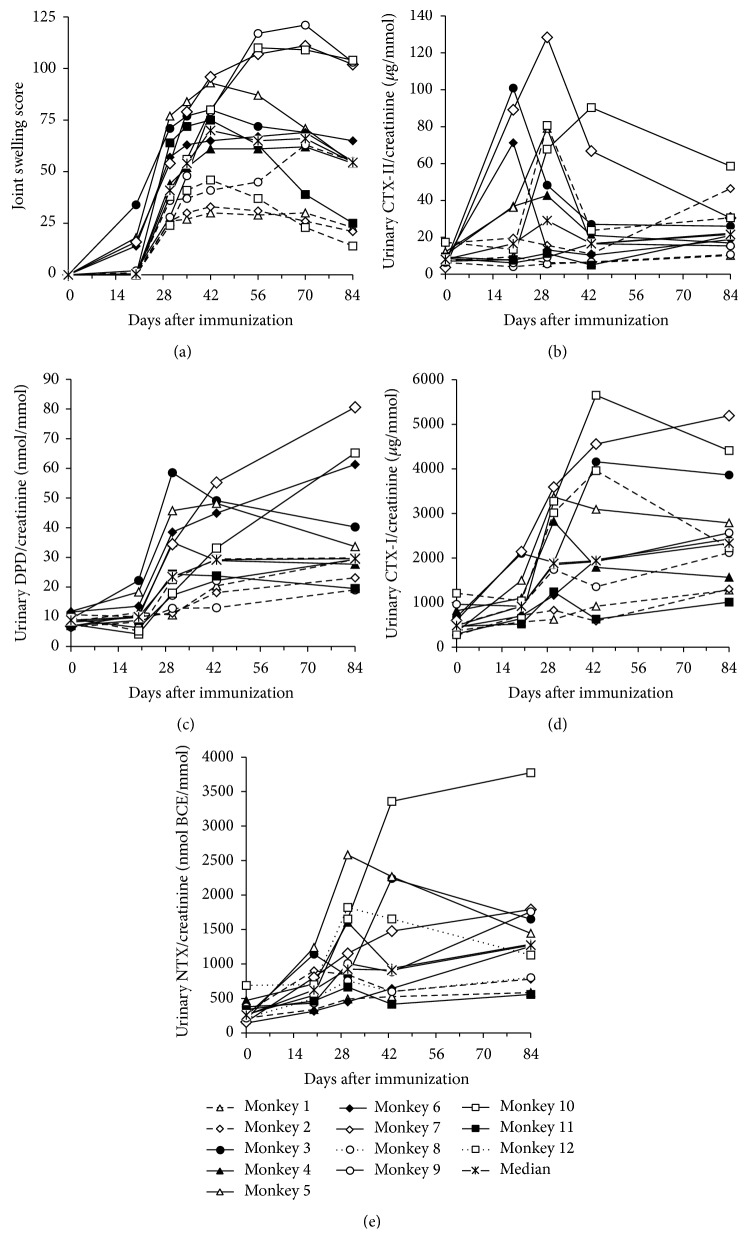
Joint swelling score, urinary cartilage marker (CTX-II/Cr), and bone turnover markers (CTX-I/Cr, NTX/Cr, and DPD/Cr).

The joint swelling score had increased in some animals on day 20 and in all animals by day 30. This score peaked on day 42 (median score of 70) and gradually decreased thereafter (Figure 1(a)). The level of a cartilage marker, urinary CTX-II/Cr, increased by a maximum of 4.4-fold (median, range 1.4–35.7) compared to the preimmunization level on day 30 and decreased thereafter (Figure 1(b)). The levels of bone turnover markers, urinary CTX-I/Cr, NTX/Cr, and DPD/Cr, had increased on day 43 and peaked on day 84 with median values of 3.7- (range 2.4–14.2), 4.2- (range 2.0–14.1), and 3.4-fold (range 2.0–9.7) those of the respective preimmunization levels (Figures 1(c)–1(e)).

**Figure 2:**
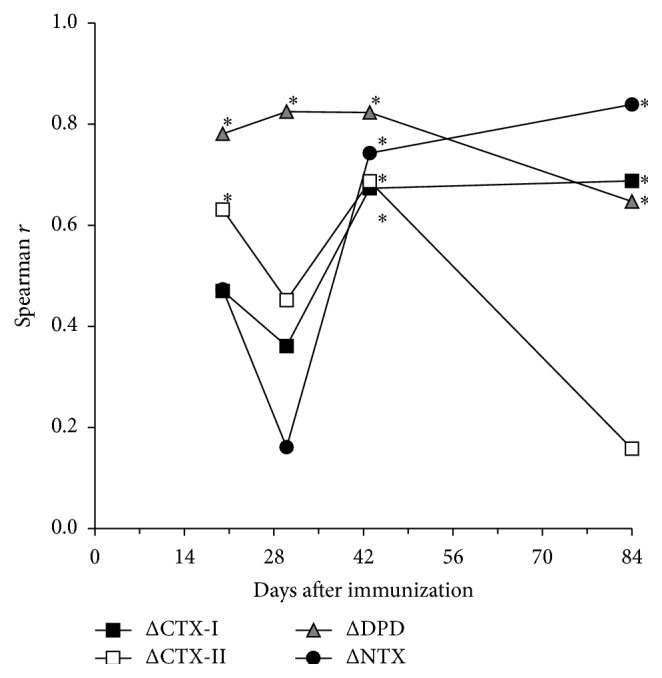
Association between joint swelling score and increases in urinary CTX-II/Cr, CTX-I/Cr, NTX/Cr, and DPD/Cr at each time point. ^*∗*^ *P* < 0.05 (Spearman rank correlation). CTX-I, C-telopeptide fragment of type I collagen; CTX-II, C-telopeptide fragment of type II collagen; DPD, deoxypyridinoline; NTX, N-telopeptide fragment of type I collagen; Cr, creatinine.

Overall correlations during study period between joint swelling score and biochemical markers based on AUCs are shown in Table 1. Changes of AUC for urinary CTX-II/Cr, CTX-I/Cr, NTX/Cr, and DPD/Cr were significantly positively correlated with the joint swelling score and other bone biochemical markers. In an analysis at each time point, the joint swelling score was positively correlated with urinary CTX-II/Cr on days 20 and 42 (Figure 2), with urinary CTX-I and NTX on days 43 and 84, and with urinary DPD/Cr at all time points.

### 3.2. Histology of Joints

**Figure 3:**
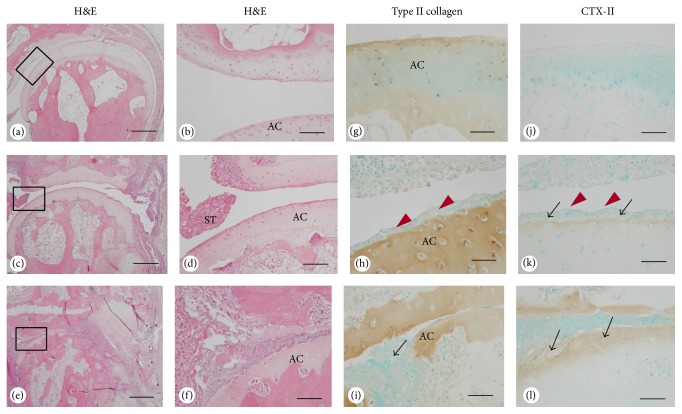
Histological analysis of sagittal sections of PIP joints of normal and arthritic monkeys. (a–f) Hematoxylin and eosin- (H&E-) stained sections of controls (a, b) and in arthritis with moderate (c, d) and severe (e, f) joint destruction. (g–l) Immunolocalization of type II collagen in controls (g) and in arthritis with moderate (h) and severe (i) joint destruction, and immunostaining of CTX-II in controls (j) and in arthritis with moderate (k) and severe (l) joint destruction. In moderate joint destruction, the superficial layer of articular cartilage showed CTX-II positivity (black arrows, k), while the deeper layer had no CTX-II reactivity. Note the unstained layer of type II collagen and CTX-II (red arrowheads, h and k). In severe joint destruction, the deeper layer of the articular cartilage became positive for CTX-II (arrows, l). Note the partial destruction of the articular cartilage (arrow, i). AC, articular cartilage; ST, synovial tissue. Bars: (a), (c), (e): 650 *μ*m, (b), (d), (f): 120 *μ*m, (g), (i), (j), (l): 100 *μ*m, and (h), (k): 50 *μ*m.

PIP joints in the 2nd finger of the left hand from 6 CIA monkeys were assessed. The joint samples were categorized as mild/none, moderate, and severe based on the gross histological classification (Table 2). PIP joint samples from 3 nonarthritic monkeys were used as controls. Histological evaluation of the PIP finger joints revealed pannus formation, expansion of synovial tissue, and inflammatory cell infiltrates extending into the joint space in arthritic monkeys (Figures 3(c)–3(f)). The pannus invaded the cartilage in joints with severe destruction. The superficial cartilage layer and the pannus invading cartilage did not stain for type II collagen in arthritic joints (Figures 3(h) and 3(i)). In contrast, the superficial layer of articular cartilage in arthritic samples was stained by CTX-II antibody, whereas CTX-II was virtually absent in nonarthritic joints (Figures 3(j)–3(l)).

**Figure 4:**
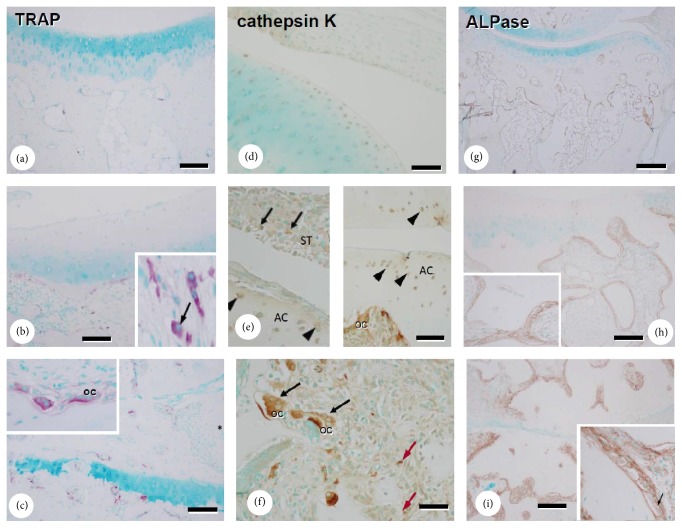
TRAP and ALPase staining and immunolocalization of cathepsin K in sagittal sections of PIP joints. TRAP staining in controls (a) and in arthritis with moderate (b) and severe (c) joint destruction. Immunolocalization of cathepsin K in controls (d) and in arthritis with moderate (e) and severe (f) joint destruction. In moderate joint destruction, the immunolocalization of cathepsin K was observed in osteoclast, cells in synovial tissue (arrows, e) and chondrocytes (arrowheads, e). In severe joint destruction, the immunolocalization of cathepsin K was observed in osteoclast (black arrows, f) and cells in connective tissue near articular joint (red arrows, f). ALPase staining in controls (g) and in arthritis with moderate (h) and severe (i) joint destruction. Black arrows, strongly cathepsin K-positive osteoclast; AC, articular cartilage; ST, synovial tissue; OC, osteoclasts. Bars: (a)–(c): 120 *μ*m, (d)–(f): 100 *μ*m, and (g)–(i): 300 *μ*m.

Immunohistological analysis of cathepsin K showed that the enzyme was abundantly expressed in TRAP-positive osteoclasts and in cells covering the articular cartilage and synovial tissues in arthritic animals (Figures 4(b), 4(c), 4(e), and 4(f)). In contrast, nonarthritic control monkeys showed faint expression in similar locations. Abundant numbers of ALPase-positive osteoblasts were present in the subchondral bone in arthritic samples, whereas controls had fewer osteoclasts and weakly stained ALPase-positive osteoblasts (Figures 4(g)–4(i)).

**Figure 5:**
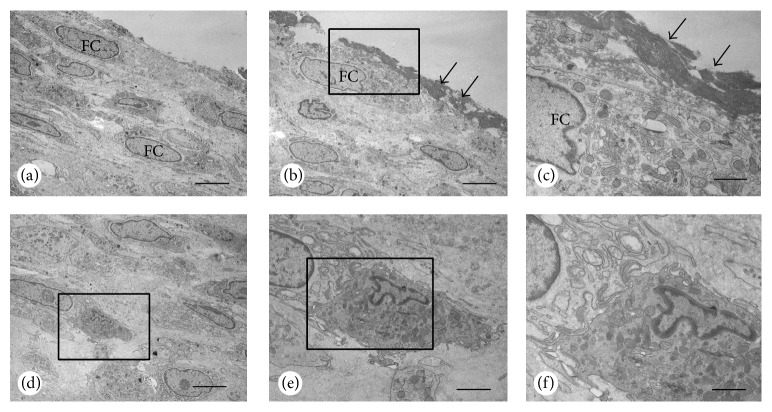
Electron microscopy of articular cartilage surface with moderate joint destruction. The surface of the articular cartilage was composed of a thick layer of fibroblastic cells (FC, a), which were sometimes covered with electron dense materials (arrows, b). At a higher magnification, electron dense materials overlaid the fibroblastic cells with abundant Golgi apparatus and rough endoplasmic reticulum (c). A macrophage can be seen in the inner portion of the fibroblastic cell layer (d). The macrophage contains many lysosomes and vesicles making contact with fibroblastic cells (e, f). Panel (f) is a magnified image of (e). Bars: (a), (b), (d): 10 *μ*m, (c), (f): 5 *μ*m, and (e): 7 *μ*m.

Interestingly, a thick cell layer covered the articular cartilage in moderate arthritic joints (Figure 5). Cellular debris overlaid this layer, but articular chondrocytes seemed to be intact. In these joints, the synovial tissues had abundant cellular debris. The cellular debris contained mainly fibroblast, but macrophages with many vesicles also existed.

## 4. Discussion

After immunization with type II collagen in cynomolgus monkeys, urinary CTX-II/Cr was increased by day 20, peaked on day 30, and decreased after day 43 (Figure 1). Joint swelling and levels of bone resorption markers (urinary NTX/Cr, CTX-I/Cr, and DPD/Cr) increased at slightly later time points than the increase in urinary CTX-II. Swelling peaked on day 30 and gradually decreased after day 43, but all bone resorption markers peaked on day 84. These data indicate that matrix degradation starts through cartilage destruction, rather than bone resorption. Similar findings have been reported in a rat CIA model [40], but the extent of the increase of CTX-II in the rat CIA model was smaller than the decrease in CTX-II with aging. AUCs of the cartilage marker (CTX-II/Cr) and all bone resorption markers were positively correlated with the joint swelling score, which suggests that matrix degradation may be regulated by the degree of joint inflammation (Table 1). NTX/Cr and CTX-I/C increases were correlated with joint swelling on days 42 and 84, and DPD/Cr showed positive correlation for all observed days in the study (Figure 2). The DPD molecule is present in bone and dentin, and also in many connective tissues, including cartilage, synovium, and muscle [41, 42]. The increases in urinary DPD/Cr on days 20 and 30 were lower than those on days 43 and 84, which might indicate less degradation of connective tissues, articular cartilage, synovium, and muscle, compared to bone. Urinary CTX-II/Cr increase was also correlated with joint swelling on days 20 and 30, and muscle wasting and necrosis of muscle fiber have been found in week 2 in a CIA model in cynomolgus monkey [34]. Thus, the increases in urinary DPD/Cr on days 20 and 30 might also be related to degradation of articular cartilage, synovium, and muscle.

An important finding in this study is that joint damage starts at the most superficial articular cartilage, and not in adjacent subchondral bone. Elevation of the cartilage marker CTX-II increased earlier than that of bone metabolic markers, CTX-I and NTX, in accord with more delayed onset of bone erosion in the CIA model. The mechanism of generation of CTX-II is not completely understood, but several proteolytic enzymes, such as matrix metalloproteinase-3 and matrix metalloproteinase-9 and cathepsins K, B, S, and L, contribute to the process in vitro [43]. Cathepsin K may have a crucial role in CTX-II generation* in vivo* because the recent observation that a cathepsin K inhibitor, SB-553484, produced a decrease in tibial cartilage degradation and a reduction in urinary CTX-I and CTX-II in a canine partial medial meniscectomy model [22]. Furthermore, another cathepsin K inhibitor, ONO-5334, decreases urinary CTX-II/Cr with a similar inhibition rate to urinary NTX/Cr in patients with osteoporosis/osteopenia [21]. A probable role of cathepsin K in cartilage degradation in aging is supported by the presence of a cathepsin K-specific neoepitope in type II collagen [44]. Cartilage proteoglycans were also cleaved by cathepsin K [45]. It is interesting to note that cartilage resident glycosaminoglycans such as chondroitin-4-sulfate enhance collagenase activity of cathepsin K by the formation of high molecular mass complexes [46, 47].

Histologically, severe joint destruction was observed in continuously swollen joints with a high joint swelling score, which suggests that joint degradation may regulate the degree of local joint inflammation (Table 2). Nonarthritic control monkeys had faint immunoreactivity for cathepsin K in cells covering articular cartilage and synovial tissues (Figure 4(d)). These results indicate that cathepsin K may have a physiological role in collagen degradation. In contrast, intense cathepsin K staining was observed at similar locations in arthritic monkeys, supporting a role for cathepsin K in cartilage degradation in the CIA model. Constitutively overexpressed cathepsin K in mice results in marked proliferative synovitis and destruction of articular cartilage and bone, and intense cathepsin K staining was detected in chondrocyte clusters in areas of cartilage degradation [18]. In a destabilization-induced mice osteoarthritis model, cathepsin K gene knockout resulted in a delay in cartilage degradation [48]. Cathepsin K expression has also been found in synovium from a patient with RA [49] and in chondroclasts in the process of endochondral ossification [50]. Thus, collectively, these results suggest that cathepsin K might have important roles in bone resorption and cartilage degradation in RA. Hou et al. reported cathepsin K expression in the RA synovium, but we demonstrated not only cathepsin K expression but also changes of cartilage as substrate, and biochemical marker of CTX-II derived from cartilage type II collagen. By addition of the evidence of cartilage degradation with changes in biochemical markers, this study provided detailed function of cathepsin K in cartilage degradation in RA.

In the current study, strong cathepsin K immunoreactivity was found in osteoclasts in CIA monkeys. Abundant TRAP-reactive and ALP-positive osteoblasts were present at the subchondral bone in arthritic joints, while controls had fewer osteoclasts and weakly stained ALP-positive osteoblasts (Figures 4(a)–4(i)). Accelerated bone turnover in arthritic monkeys was confirmed by biochemical markers and histological analysis. In immunohistological analysis (Figure 3), CTX-II was detected in the superficial layer of articular cartilage in arthritic joints but was virtually absent in controls. In contrast, the superficial cartilage layer and the pannus that invaded the cartilage area did not stain for type II collagen. CTX-II is neoepitope of type II collagen generated by cathepsin K involved proteases. The loss of staining for type II collagen in the superficial cartilage layer and the pannus invading cartilage in arthritic joints was demonstrated to couple to increase in CTX-II staining. These histological changes might relate to cartilage destruction by cathepsin K. A thick cell layer covered the articular cartilage in arthritic joints and cellular debris overlaid this layer, but articular chondrocytes were intact, while synovial tissues included extensive cellular debris (Figure 5). The findings were matching light microscope findings (see Supplemental Figure in the Supplementary Material available online at http://dx.doi.org/10.1155/2016/8938916). The changes were not only deposit of organic substances but also thick cell layer; mainly fibroblasts were observed. Thus, fibroblasts covering the articular surface might be observed in early stages of arthritis.

## 5. Conclusions

In summary, a key finding in this study of longitudinal changes in a monkey CIA model was that joint damage starts with cartilage degradation in the most superficial layer of articular cartilage, rather than through bone resorption, and is coupled with a transient increase in the urinary cartilage marker CTX-II. The expression of cathepsin K was found in articular cartilage and synovial tissue, as well as in osteoclasts, which suggests that cathepsin K may have roles in both bone resorption and degradation of cartilage in the monkey CIA model. In addition, the loss of staining for type II collagen in the superficial cartilage layer and the pannus invading cartilage in arthritic joints seems to couple to increase in CTX-II staining. The evidence in this study indicates that matrix degradation may start from the most superficial region of the arthritic cartilage, as well as the adjacent subchondral bone.

## Supplementary Material

Synovium was thickened, and organic substances were deposited on articulating surface.

## Figures and Tables

**Table 1:** Correlations between area under the concentration/score curve of changes in cartilage marker, bone turnover markers, and joint swelling score.

	Spearman *r*			

AUC of markers	ΔCTX-I/Cr (AUC)	ΔCTX-II/Cr (AUC)	ΔDPD/Cr (AUC)	ΔNTX/Cr

Joint swelling score (AUC)	0.673^*∗*^	0.687^*∗*^	0.823^*∗*^	0.743^*∗*^

Correlation coefficients are shown. ^*∗*^ *P* < 0.05 (Spearman rank correlation); CTX-I, C-telopeptide fragment of type I collagen; CTX-II, C-telopeptide fragment of type II collagen; DPD, deoxypyridinoline; NTX, N-telopeptide fragment of type I collagen; Cr, creatinine; AUC, area under the concentration/score-versus-time curve.

**Table 2:** Background data for joint samples.

Histological classification	Mild/none			Moderate		Severe

Histological findings	No change to nonarthritic joint			Fibrillation of articular cartilage^$^, surrounding soft tissue growth		Destruction of joint, invasion of soft tissue into osseous tissue

Animal number	3	6	8	4	9	5

Maximum joint swelling score, all joints (day)	28 (49)	48 (49)	20 (49)	65 (49)	61 (49)	87 (35)

Joint swelling score of distal interphalangeal (DIP) joint^*∗*^						
Day 21	0	0	0	0	0	0
Day 35	0	0	0	1	0	1
Day 49	0	0	0	1	0	2
Day 63	0	0	0	1	0	2

Joint swelling score of adjacent proximal interphalangeal (PIP) joint^*∗*^						
Day 21	1	0	0	1	0	3
Day 35	1	0	0	3	2	3
Day 49	2	0	1	3	3	3
Day 63	2	0	1	3	3	3

^*∗*^The 2nd finger of the left hand

^$^Rigidity of the joints

Joint swelling score: 0, no abnormality; 1, swelling not visible but can be determined by touch; 2, swelling slightly visible and can be confirmed by touch; 3, swelling clearly visible; 4, rigidity of the joints.

## Abbreviations

Cr: Creatinine
CTX-I: C-telopeptide of type I collagen
CTX-II: C-telopeptide of type II collagen
DPD: Deoxypyridinoline
NTX: N-telopeptide of type I collagen
MMP-1: Matrix metalloproteinase-1
SE: Standard error.
