# Supplementary material for: Joint Degradation in a Monkey Model of Collagen-Induced Arthritis: Role of Cathepsin K Based on Biochemical Markers and Histological Evaluation
Source: Int J Rheumatol. 2016 Feb 2;2016:8938916. doi: 10.1155/2016/8938916 (PMC4754492; doi:10.1155/2016/8938916)
Supplement: Supplementary file 1 — Synovium was thickened, and organic substances were deposited on articulating surface. [file 8938916.f1.pdf]

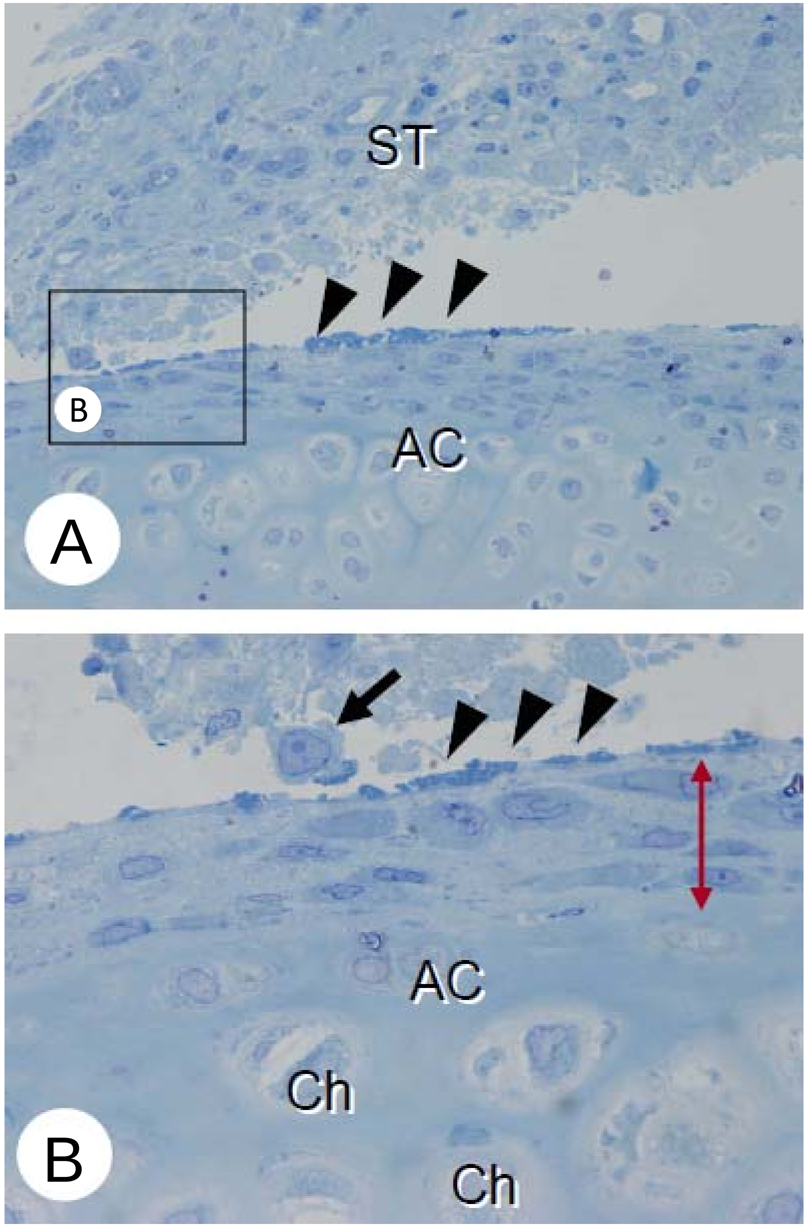

Supplement Figure. Light microscopy of articular cartilage surface with moderate joint destruction. The framework of Fig. B was showed in Fig. A. Synovium was thickened, and organic substances were deposited on articulating surface (Arrowheads: A, B). Mononuclear cell was localized into joint space (Arrow: B). Cells in articular surface were not oval chondrocytes but multiple layers of flat cells (Red arrow). AC: articular cartilage ST: synovial tissue Ch: chondrocytes
